# High-Intensity Interval Training in Older Adults: a Scoping Review

**DOI:** 10.1186/s40798-021-00344-4

**Published:** 2021-07-19

**Authors:** Catherine F. S. Marriott, Andrea F. M. Petrella, Emily C. S. Marriott, Narlon C. Boa Sorte Silva, Robert J. Petrella

**Affiliations:** 1 Centre for Studies in Family Medicine, Department of Family Medicine, Schulich School of Medicine and Dentistry, Western University, London, ON Canada; 2 Aging, Mobility, and Cognitive Neuroscience Lab, Djavad Mowafaghian Centre for Brain Health, University of British Columbia, Vancouver, BC Canada; 3 Department of Physical Therapy, Faculty of Medicine, University of British Columbia, Vancouver, Canada; 4 School of Kinesiology, Western University, London, ON Canada; 5 School of Kinesiology, Faculty of Education, University of British Columbia, Vancouver, BC Canada; 6 Department of Family Practice, Faculty of Medicine, University of British Columbia, 320 - 5950 University Boulevard, Vancouver, BC V6T 1Z3 Canada

**Keywords:** High-intensity interval training, Older adults, Seniors, Chronic disease

## Abstract

High-intensity interval training (HIIT) is an increasingly popular form of aerobic exercise which includes bouts of high-intensity exercise interspersed with periods of rest. The health benefits, risks, and optimal design of HIIT are still unclear. Further, most research on HIIT has been done in young and middle-aged adults, and as such, the tolerability and effects in senior populations are less well-known. The purpose of this scoping review was to characterize HIIT research that has been done in older adults including protocols, feasibility, and safety and to identify gaps in the current knowledge. Five databases were searched with variations of the terms, “high-intensity interval training” and “older adults” for experimental or quasi-experimental studies published in or after 2009. Studies were included if they had a treatment group with a mean age of 65 years or older who did HIIT, exclusively. Of 4644 papers identified, 69 met the inclusion criteria. The average duration of training was 7.9 (7.0) weeks (mean [SD]) and protocols ranged widely. The average sample size was 47.0 (65.2) subjects (mean [SD]). Healthy populations were the most studied group (*n* = 30), followed by subjects with cardiovascular (*n* = 12) or cardiac disease (*n* = 9), metabolic dysfunction (*n* = 8), and others (*n* = 10). The most common primary outcomes included changes in cardiorespiratory fitness (such as VO_2peak_) as well as feasibility and safety of the protocols as measured by the number of participant dropouts, adverse events, and compliance rate. HIIT protocols were diverse but were generally well-tolerated and may confer many health advantages to older adults. Larger studies and more research in clinical populations most representative of older adults are needed to further evaluate the clinical effects of HIIT in these groups.

**Supplementary Information:**

The online version contains supplementary material available at 10.1186/s40798-021-00344-4.

## Key Points

- High-intensity interval training, though increasingly popular, has not been well-studied in older adults.
- Early research suggests that HIIT may confer health benefits over moderate-intensity continuous training (traditional endurance exercise) and is generally well-tolerated in older adults.

## Introduction

Globally, the number of people aged 65 years or older is expected to more than double in the next 30 years, making it the fastest growing age demographic [1]. It has been estimated that these older adults experience 23% of the global burden of disease and that this number increases to nearly 50% in high-income countries and is about 20% in low- and middle-income countries [2]. Chronic non-communicable diseases make up the majority of this burden [2]. This accounts for a significant and growing financial burden on our health care systems and the UN National Assembly (2012) has acknowledged the urgent need for governments to scale up and transition towards universal, affordable, and quality health-care services [3]. Exercise is known to be an important part of healthy aging and is useful in preventing and managing chronic disease [4]. The Physical Activity Guidelines for Americans [5] recommend that adults over 65 years of age achieve at least 150 min of moderate- or 75 min of vigorous-intensity aerobic physical activity per week, in addition to muscle- and bone-strengthening activities at least 2 days per week. They also highlight that in this population, exercise can be done to improve both health outcomes and functional abilities and that they should include multicomponent training that incorporates balance and flexibility training [5]. As such, identifying modes of exercise which achieve these goals and are tolerable and feasible in older adults is an important step towards improving the health of these populations.

High-intensity interval training (HIIT) is an increasingly popular form of aerobic exercise which includes bouts of high-intensity exercise, typically lasting seconds to minutes, interspersed with periods of rest [6]. HIIT has been proposed to be equal or advantageous to continuous endurance training both in terms of physiologic results [7, 8] and in enjoyability [9]. However, the health benefits, risks, and optimal design of HIIT are still unclear. Further, most of the research on the effects and benefits of HIIT has been done in younger and middle-aged adults, and as such, the tolerability and effects in older populations are less well-known.

The purpose of this scoping review is to identify and characterize existing research on the effects of HIIT in older adults to assist in knowledge translation and recommend further areas of study. Specifically, it aims to describe which study populations are included, the training protocol designs, whether this training is feasible and/or tolerable for older adults, the main outcomes being addressed, and to identify gaps in the current knowledge.

## Methodological Framework

According to Arksey and O’Malley [10], the purpose of a scoping review is to examine the extent, range, and nature of research activity, to summarize research findings, or to identify gaps in the existing literature. To achieve this, they established a 5-step framework which was used in the undertaking of this review. The steps are detailed below and include the following: 1. Identifying the research question, 2. Identifying relevant studies, 3. Study selection, 4. Charting the data, 5. Collating, summarizing, and reporting the results [10].

The research question was as follows: What is known in the literature about HIIT in older adults, including which protocols are used, outcomes measured, its feasibility and safety in this population, and what are the gaps in the current knowledge?

A full description of the study protocol including search strategy and detailed reasons for article exclusion are available in the supplemental materials. In summary, five databases were searched (Scopus, Medline (Ovid), Embase (Ovid), CINAHL, and SportDiscus) for articles published up to February 2021. Search terms included combinations and variations of the following: “high-intensity interval training,” “interval aerobic training,” “HIIT,” “older adult,” and “senior.” A description of the complete search strategy is included in the supplemental materials*.* These searches identified 4644 potential studies. Of these, 2019 references were removed as duplicates. The non-duplicate titles and abstracts were read by authors CM and AP to determine if the studies were relevant to the research question. Initial exclusion criteria were as follows: (1) published before 2009 (as it was not feasible for the authors to screen all of the potential studies and the authors wanted to include the most recent and relevant studies), (2) review papers or not peer-reviewed, (3) did not include high-intensity exercise protocols, (4) did not use human subjects, or (5) the mean age of the study subjects was less than 50 years old.

In keeping with the iterative nature of scoping review methods, inclusion criteria were then developed in collaboration with author RP. The inclusion criteria were as follows: (1) the mean age of all participants was at least 65 years of age or older, or one mean cohort age was at least 65 years and was not statistically different from the other groups, (2) the study was an experimental or semi-experimental trial, (3) the study was an original source (for example, letters to the editor, correspondences, and editorials were not included) published as full-text in English, and (4) the exercise protocol used was exclusively high-intensity interval aerobic training, and was not combined with another intervention, such as resistance training (RT). Many exercise training modalities which have some similarities to HIIT were excluded from this review. These included RT or high-intensity resistance training, which primarily aims to overload the musculoskeletal system by causing the muscles to contract against an external force [11], circuit training and body-weight interval training (which includes RT), moderate-intensity interval training (MIT), and moderate-intensity continuous training (MCT). Additionally, high-intensity functional exercise is a form of functional weight-bearing exercise training designed for the elderly populations dependent on activities of daily living. This type of training more closely resembles RT than HIIT, and as such, it was also excluded from this review [12]. Exercise intensity is often measured using heart rate (HR), heart rate reserve (HRR), or oxygen uptake (VO_2_). “High-intensity” was defined and categorized as vigorous effort (70–89% of peak HR; 60–84% of HRR; 60–79% of peak VO_2_) or “very hard” effort (≥ 90% of peak HR; ≥ 85% of HRR; ≥ 80% of peak VO_2_) [13, 14]. The anaerobic threshold (VT_2_) was included as high-intensity, but the aerobic threshold (VT_1_) was not [15]. The percentage of peak power output (%PPO) is occasionally used to measure and report exercise intensity. There are reports that %PPO does not correlate with the same percentage of HR_max_ in different exercise modalities/patient populations [16]. To illustrate this point, Hood et al. [17] used a target intensity of 60% PPO which correlated with approximately 80% HRR and increased over time to 95% HRR. Where a range of target intensities was described in the study protocols, the average intensity was used to determine eligibility. For example, if the study had participants exercise at 60–70% HR_peak_, it was excluded as the average intensity was presumed to be 65% HR_peak_. Sprint interval training (SIT) is an interval exercise involving maximal or supramaximal intensity activity for short periods of time (typically seconds) [7]. Though different from HIIT, SIT treatment groups were included in this review as they may offer further insight into the tolerability, safety, and acceptance of similar interval protocols. The remaining articles were read in full by authors CM and AP to assess for eligibility. Any discrepancy was discussed by these authors until consensus was achieved.

The eligible studies were read and grouped by clinical populations. Data from these studies were extracted and charted by CM, including the population(s) studied, the study design, and the main outcomes measured. Details of the HIIT protocol intervention were also charted and included exercise frequency, intensity and duration of interval, intensity and duration of the rest period, and modality (such as treadmill, cycling, etc.). If it was noted in the publication, information on whether the HIIT was feasible and/or tolerated by study participants was also extracted. This was done by measuring outcomes such as attendance, adherence, drop-outs/withdrawals, “enjoyability” or acceptance of protocol, and adverse events. These data were validated by research assistant, EM. In charting the study design, the HIIT interventions were summarized to allow for ease of comparison between studies. Controls or other treatment groups compared to HIIT in the literature were noted and included RT and MCT. MCT was defined as an intensity of 55–69% HR_max_ or 40–59% VO_2max_ and is representative of typical endurance training [13]. RT was defined as exercise primarily aiming to overload the musculoskeletal system by causing the muscles to contract against an external force [11]. Data were summarized and reported as per the emerging themes.

## Results

**Fig. 1:**
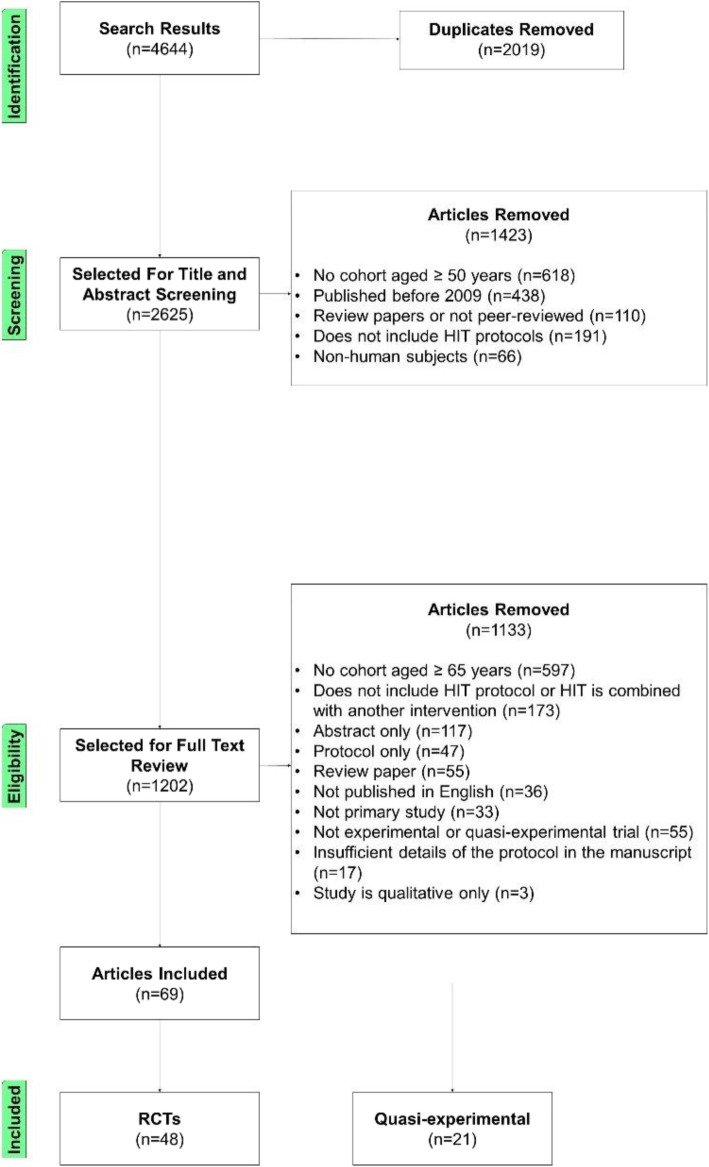
Flow chart of the study selection process

The search yielded 4644 references. Duplicates were removed and inclusion and exclusion criteria were applied. Age was a common reason for exclusion. As such, if the mean age was not provided in the abstract, this information was found in the full text as part of the screening process. In the papers assessed for eligibility, resistance or circuit training were often combined with HIIT. However, as many papers focused exclusively on HIIT, these combined programs were excluded from the final subset. This left 69 studies to be included in the review (Fig. 1).

The studies included were classified by clinical population. The largest grouping was of non-clinical populations (*n* = 30), followed by cardiovascular diseases (*n* = 12), cardiac disease (*n* = 9), metabolic disease (*n* = 8), and other (*n* = 10). Study design, sample size, population(s) included, and baseline characteristics of included studies, grouped by clinical cohort, are reported in Table 1. These studies had sample sizes ranging from 10 to 473 (mean [SD] = 47.0 [65.2]) and included a total of 3243 individuals. The mean ages of the study participants ranged from 61.4 to 80.8 years (mean [SD] = 67.9 [3.4] years). Forty-eight studies were randomized controlled or crossover designs; 21 were quasi-experimental.

**Table 1:** Study design, sample size, and participant characteristics of included studies grouped by clinical population

Study	Study design	Population	Sample N	Age (years ± SD)	Females N (%)
Non-clinical populations					
Aboarrage et al. (2018) [18]	RCT; HIIT vs control^a^	Sedentary, healthy women	25	65 ± 7	25 (100)
Adamson et al. (2019) [19]	QE; SIT vs control^a^	Sedentary, with well-controlled HTN, taking oral anti-hypertensive medication	17	66 ± 3	8 (47)
Bailey et al. (2017) [20]	RCT (crossover); HIIT vs MCT vs control^b^	Higher-and lower-fit healthy males	47	70 ± 5	0 (0)
Brown et al. (2021) [21]	RCT; HIIT vs MCT vs control^b^	Cognitively normal older adults	99	69.1 ± 5.2	54 (55)
Bruseghini et al. (2015) [22]	QE (within-subject); HIIT vs RT	Moderately active males	12	68 ± 4	0 (0)
Bruseghini et al. (2020) [23]	RCT; HIIT vs MCT	Healthy active older males	24	69.6 ± 4.1	0 (0)
Coswig et al. (2020) [24]	RCT; HIIT vs MCT vs MIIT	Sedentary, female residents of a nursing home without comorbidities that would preclude participation	46	80.8 ± 5.2	46 (100)
Donath et al. (2015) [25]	QE; HIIT	Healthy and physically active older and young adults	40	70 ± 4, 27 ± 3	21 (53)
Herrod et al. (2020a) [26]	QE; HIIT (2-, 4-, or 6-week intervention) vs control^b^	Healthy, recreationally active older adults	40	71 ± 5	19 (48)
Herrod et al. (2020b) [27]	RCT: HIIT vs isometric handgrip training vs remote ischemic preconditioning vs control^b^	Healthy older adults	48	71 ± 4	22 (46)
Hwang et al. (2016) [28]	RCT; HIIT vs MCT vs control^b^	Sedentary older adults	43	65 ± 1	–
Kim et al. (2017) [29]	RCT; HIIT vs MCT vs control^b^	Healthy, sedentary adults	49	64 ± 1	–
Kovacevic et al. (2020) [30]	RCT; HIIT vs MCT vs stretching	Sedentary, healthy older adults	64	72 ± 5.7	39 (61)
Krusnauskas et al. (2018) [31]	RCT (crossover); SIT (6 × 5 s or 3 × 30 s “all-out” vs 3 × 60 s “submaximal”)	Young and older women	19	65.7 ± 2.8, 19.5 ± 1.3	19 (100)
Linares et al. (2020) [32]	RCT (crossover); HIIT vs MCT vs SIT	Healthy older adults recruited from cycling clubs and recreational centers	30	69.6 ± 6.2	15 (50)
McSween et al. (2020) [33]	RCT; HIIT vs MCT vs stretching	Healthy older adults	60	66.4 ± 4.6	43 (72)
Mejias-Pena et al. (2016) [34]	RCT; HIIT vs control^a^	Healthy older adults	29	69.7 ± 1	21 (72)
Mekari et al. (2020) [35]	RCT; SIT vs MCT vs RT	Healthy, active older adults	69	68 ± 7	42 (61)
Nakajima et al. (2010) [36]	QE; HIIT vs control^c^	Older participants in a health promotion program and young controls	473	65.4 ± 7.5, 19.4 ± 0.9	–
Nederveen et al. (2015) [37]	RCT; HIIT vs MCT vs RT	Sedentary older men	22	67 ± 4	0 (0)
O’Brien et al. (2020) [38]	RCT; SIT vs MCT vs RT	Healthy, active older adults	38	67 ± 6	23 (61)
Osuka et al. (2017) [39]	QE (within-subject); HIIT vs MCT	Elderly men	21	67.6 ± 1.8	0 (0)
Stockwell et al. (2012) [40]	RCT (crossover); HIIT vs MCT	Participants with baseline exercise of 90 min per week	22	68.4 ± 3.8	6 (38)
Storen et al. (2017) [41]	QE; HIIT	Healthy adults (ages 20–83 y) divided into age cohorts by decades	94	70+ cohort: 74.4 ± 4.4	22 (23)
Venckunas et al. (2019) [42]	RCT (crossover); SIT (6 × 5s or 3 × 30 s “all-out” vs 3 × 60 s “submaximal”)	Untrained young, endurance-trained young cyclists, and untrained older males	11	69.9 ± 6.3	0 (0)
Vogel et al. (2011) [43]	QE; HIIT	Untrained “older” and “young” seniors	150	66.0 ± 6.9	70 (47)
Windsor et al. (2018) [44]	RCT (crossover); HIIT vs MCT vs control^b^	Lower-fit and higher-fit healthy older adults	30	70.6 ± 5.7	4 (13)
Wyckelsma et al. (2017) [45]	QE; HIIT vs control^b^	Older adults, active at baseline	15	69.4	6 (40)
Yasar et al. (2019) [41]	RCT (crossover); SIT (interspersed with 3 or 5 days of recovery)	Physically active older and young adults	18	70 ± 8, 24 ± 3	6 (33)
Yoo et al. (2017) [46]	RCT (crossover); HIIT vs MCT vs LCT	Healthy men and post-menopausal women	28	67 ± 1	15 (54)
Cardiovascular populations					
Bailey et al. (2018) [47]	RCT (crossover); HIIT vs MCT vs control^b^	Males, healthy or with AAA	44	73 ± 6	0 (0)
Currie et al. (2012) [48]	RCT (crossover); HIIT vs MCT	Participants with CAD	10	66 ± 1	1 (10)
Currie et al. (2013) [49]	RCT; HIIT vs MCT	Participants with a recent CAD event	22	65 ± 10	2 (9)
dos Santos et al. (2018) [50]	RCT (crossover); HIIT vs MCT	Participants with HTN	15	65.1 ± 5.37	–
Guiraud et al. (2009) [51]	RCT (crossover); SIT (15s or 60-s intervals with passive or active rest)	Participants with stable CAD	19	65 ± 8	2 (11)
Helgerud et al. (2009) [52]	QE; HIIT vs control^e^	Participants with peripheral arterial disease	21	67.5 ± 6.3	4 (19)
Moore et al. (2020) [53]	QE (pre-post); HCT vs control^d^	Stroke rehabilitation inpatients	110	73.5 ± 12.2	47 (43)
Nepveu et al. (2017) [54]	RCT; HIIT vs control^b^	Patients with chronic stroke, average MoCA = 25.3	22	64.9 ± 11.2	5 (23)
Reichert et al. (2016) [55]	RCT; HIIT vs HCT (both with stretching)	Participants with HTN	25	67.9 ± 5.9	–
Sosner et al. (2016) [56]	RCT; HIIT (dryland vs immersed) vs MCT	Participants with HTN	42	65 ± 7	20 (48)
Tew et al. (2017) [57]	RCT; HIIT vs usual care for 4 weeks before surgery	Participants with infrarenal AAA who were eligible for open or endovascular repair	53	74.7 ± 5.9	3 (6)
Windsor et al. (2018) [58]	RCT (crossover); HIIT vs MCT vs control^b^	Healthy or with small AAAs	40	72.5 ± 5.7	0 (0)
Cardiac disease					
Angadi et al. (2015) [59]	RCT; HIIT vs MCT	Patients with HFpEF and NYHA II-III	15	70 ± 8.3	3 (20)
Ellingsen et al. (2017) [60]	RCT; HIIT vs MCT vs control^e^	Patients with LVEF ≤ 35% and NYHA II-III	231	61.8	40 (17)
Fu et al. (2013) [61]	RCT; HIIT vs MCT vs control^d^	Participants with HF	45	67.2 ± 2.2	16 (36)
Iellamo et al. (2014) [62]	RCT; HIIT vs MCT	Chronic HF secondary to CAD	36	67.8 ± 7.0	5 (14)
Isaksen et al. (2015) [63]	QE; HIIT vs control^b^	Participants with HF and an implantable defibrillator	35	66.2 ± 9.1	3 (8)
Isaksen et al. (2016) [64]	QE; HIIT vs control^b^	Participants with ischemic heart disease and an implantable cardioverter defibrillator	30	67.1 ± 9.0	2 (7)
Munch et al. (2018) [65]	QE; HIIT	Healthy or patients with HF	14	61.4 ± 5.2	2 (25)
Spee et al. (2020) [66]	RCT; HIIT vs control^d^	Participants with HF selected for cardiac resynchronization therapy	24	68.9 ± 6.4	5 (21)
Thijssen et al. (2019) [67]	QE; HIIT vs MCT vs control^b^	Participants with HF	29	65 ± 8	5 (17)
Metabolic disease					
Andonian et al. (2018) [68]	QE; HIIT	Sedentary patients with prediabetes or rheumatoid arthritis	21	Prediabetes: 71.4 ± 4.9 Rheumatoid Arthritis: 63.9 ± 7.2	16 (76)
Bartlett et al. (2020) [69]	QE; HIIT	Sedentary older adults with prediabetes and healthy young adults	10	71 ± 5	6 (60)
Boukabous et al. (2019) [70]	RCT; HIIT vs MCT	Women with abdominal obesity	18	65.1 ± 3.6	18 (100)
Hwang et al. (2019) [71]	RCT; HIIT vs MCT vs control^b^	Participants with T2DM	50	63 ± 1	23 (46)
Karstoft et al. (2017) [72]	RCT (crossover); MCT vs HIIT vs control^b^	Participants with T2DM	14	65.3 ± 1.7	3 (21)
Maillard et al. (2016) [73]	RCT; HIIT vs MCT	Overweight women with T2DM	17	69 ± 1	17 (100)
Mohammadi et al. (2017) [74]	QE; HIIT vs control^a^	Obese men	24	71.6 ± 5.0	0 (0)
Pandey et al. (2017) [75]	RCT; HCT vs MCT	Participants newly diagnosed with T2DM	40	66.6 ± 9.0	12 (30)
Other					
Banerjee et al. (2018) [76]	RCT; HIIT vs control^d^	Participants with bladder cancer listed for radical cystectomy	60	72.1 ± 7.6	7 (12)
Devin et al. (2019) [77]	QE; HIIT (single session vs 4-week training)	Male colorectal cancer survivors	20	65.9 ± 7.2	0 (0)
Fiorelli et al. (2019) [78]	RCT (crossover); HIIT vs MCT vs control^b^	Participants with Parkinson’s disease	12	66.5 ± 8.0	6 (50)
Hoffmann et al. (2016) [79]	RCT; HIIT vs control^a^	Community-dwelling participants with mild Alzheimer’s disease	200	70.5 ± 7.4	87 (44)
Keogh et al. (2018) [80]	RCT; MCT vs HIIT	Participants with knee osteoarthritis	17	62.4 ± 8.3	13 (76)
Mitropoulos et al. (2018) [81]	RCT; SIT (arm crank or cycling) vs control^b^	Participants with limited cutaneous systemic sclerosis	34	65.3 ± 11.6	31 (91)
Northey et al. (2019) [82]	RCT; HIIT vs MCT vs control	Breast cancer survivors	17	62.9 ± 7.8	17 (100)
Rizk et al. (2015) [83]	RCT; HIIT vs HCT vs MCT	Participants with COPD	35	67.3 ± 8.8	21 (60)
Rodriguez et al. (2016) [84]	QE; HIIT vs MCT	Participants with COPD	29	68 ± 8	2 (7)
Uc et al. (2014) [85]	QE (initially randomized, then all allocated to MCT only); HIIT vs MCT and individual vs group training	Participants with Parkinson’s disease, Hoehn and Yahr stages 1–3	60	65.4 ± 6.2	19 (31.7)

Control specifiers: ^a^usual activities; ^b^non-exercise control; ^c^type of control not specified; ^d^usual healthcare; ^e^recommendation of usual exercise

*RCT* randomized controlled trial (if not further specified, parallel design); *QE* quasi-experimental (if not further specified, parallel design); *HIIT* high-intensity interval training; *SIT* sprint interval training; *HCT* high-intensity continuous training; *MCT* moderate-intensity continuous training; *LCT* low-intensity continuous training; *RT* resistance training; *HTN* hypertension; *AAA* abdominal aortic aneurysm; *CAD* coronary artery disease; *HF* heart failure; *HFpEF* heart failure with preserved ejection fraction; *NYHA* New York Heart Association; *LVEF* left ventricular ejection fraction; *T2DM* type 2 diabetes mellitus; *COPD* chronic obstructive pulmonary disease

Tables 2, 3, 4, 5, and 6 show the HIIT protocols, outcomes, and feasibility/tolerability (where available) of studies grouped by clinical population. Across all clinical populations, the interval training interventions ranged from a single session (*n* = 14) to 6 months (*n* = 4) of training (mean [SD] = 7.9 [7.0] weeks). In non-acute training interventions, session frequency ranged from 2 to 5 training sessions per week. The training interventions included SIT (*n* = 12), and intervals defined by Buchheit and Laursen [86] as short (< 1 min) (*n* = 3), and long (≥ 1 min) (*n* = 57) in duration. The most common modality for achieving HIIT was to use a cycle ergometer (*n* = 46) followed by treadmills/walking, water-based aerobic training, all-extremity non-weight-bearing ergometers, and recumbent steppers. Most training interventions measured the intensity using HR or VO_2_ achieved (as percentage of HR_peak_, HR_max_, VO_2peak_, or VO_2max_). However, some used percentage of PPO or work (W) (W_peak_, W_max_) as metrics. Where it was reported, most authors agreed that the HIIT intervention was generally well-tolerated by study participants. The protocols used, outcomes, and feasibility findings were further examined by clinical groups. Trends seen are discussed below.

**Table 2:** HIIT studies in non-clinical populations

Article	HIIT/SIT protocol	Outcomes	Feasibility/tolerability
Aboarrage et al. (2018) [18]	Frequency: 3×/week for 24 weeks Intervals: 20 bouts at “all-out” intensity for 30-s Rest: 30-s passive recovery Time: 20 min Modality: Jump-based aquatic training	There was a significant increase in bone mineral density of the lumbar spine, total femur, and whole body of HIIT compared to the control group. Functional ability was also improved in HIIT compared to control as measured by the timed up-and-go test (improved by − 11 ± 4%) and Chair Stand (improved by 17 ± 3%).	Dropouts: None reported AEs: None reported
Adamson et al. (2019) [19]	Frequency: 2×/week for 10 weeks Intervals: 6–10 intervals of “all-out” or submaximal intensity for 6s Rest: 1-min passive recovery Time: 12 min max Modality: Cycle ergometer	Compared to control there was a significant decrease in SIT group in systolic (7%), diastolic (9%), pulse pressure (9%), and MAP (8%) as well as improvement in physical function (timed up and go, loaded 50m walk, and stair climb power). Additionally, ratios of total cholesterol/HDL cholesterol and LDL cholesterol/HDL cholesterol were significantly reduced compared to control after SIT.	Dropouts: None Compliance: 100% completion rate by all participants. AEs: None reported
Bailey et al. (2017) [20]	Frequency: Single session Intervals: 12 intervals at 70% PPO for 1 min Rest: 1 min at 10% PPO Modality: Cycle ergometer	After MCT there was an immediate increase in FMD that normalized after 1h in both fitness groups. After HIIT, FMD decreased immediately and 1 h post-intervention in the lower fit group but increased after 1h in the higher-fit group.	Not provided
Brown et al. (2021) [21]	Frequency: 2×/week for 6 months Intervals: 11 intervals at 18 RPE on Borg scale for 1 min Rest: 2-min active recovery Modality: Cycle ergometer	The HIIT group experienced greater increases in fitness than the moderate-intensity and control groups. However, there was no direct effect of exercise on cognition.	Compliance: No difference in exercise attendance between HIIT (85.5 ± 12.4%) and MCT (86.3 ± 9.8%) AEs: No serious AEs recorded Dropouts: 7 withdrew during the intervention period (HIIT: 1 due to medical illness and 1 due to pain after exercise; MCT: 2 due to medical illness and 1 due to refusing participation; control: 1 due to medical illness and one refused participation)
Bruseghini et al. (2015) [22]	Frequency: 3×/week for 8 weeks Intervals: 7 intervals at 85–95% VO_2max_ for 2 min Rest: Active recovery at 40% VO_2max_ for 2 min Modality: Cycle ergometer	Cardiovascular fitness significantly improved and systolic BP decreased in the HIIT group. Both HIIT and RT resulted in quadriceps hypertrophy but there was only an associated increase in strength after RT.	Not provided
Bruseghini et al. (2020) [23]	Frequency: 3×/week for 8 weeks Intervals: 7 intervals at 85–95% VO_2max_ for 2 min Rest: 2 min at 40% VO_2max_ Modality: Cycle ergometer	During HIIT, significant changes were observed in moderate and vigorous physical activity, average daily metabolic equivalents (METs), physical activity level, and activity energy expenditure (*p* < 0.05) but not in total energy expenditure. Sleep and sedentary time, and levels of light physical activity remained constant.	Not given
Coswig et al. (2020) [24]	Frequency: 2×/week for 8 weeks Intervals: 4 intervals at 85–95% HR_max_ for 4 min Rest: 4 min at 65% HR_max_ Modality: Treadmill	HIIT promoted greater reductions in body mass (HIIT = − 1.6 ± 0.1 kg; MICT = − 0.9 ± 0.1 kg; MIIT = − 0.9 ± 0.1 kg; *p* = 0.001), fat mass (HIIT = − 2.2 ± 0.1%; MICT = − 0.7 ± 0.1%; MIIT = − 1.2 ± 0.1%; p<0.001), resting heart rate (HIIT = − 7.3 ± 0.3%; MICT = − 3.6 ± 0.3%; MIIT = − 5.1 ± 0.3%; *p* < 0.001) and greater improvement in the chair stand test.	Dropouts: None
Donath et al. (2015) [25]	Frequency: Single session Intervals: 4 intervals at 90–95% HR_max_ for 4 min Rest: 3 min at 70% HR_max_ Modality: Treadmill	Standing balance performance: seniors demonstrated inverted ankle muscle coordination pattern compared to young adults which was unchanged by HIIT. Ankle co-activation was twofold elevated in seniors compared to young adults during single limb stance with eyes open and was also not affected by HIIT.	Not given
Herrod et al. (2020a) [26]	Frequency: 3×/week for 2, 4, or 6 weeks Intervals: 5 intervals at 90–110% of PPO for 1 min Rest: 90 s of active recovery Modality: Cycle ergometer	Anaerobic threshold was increased only after 4 (+1.9 ± 1.1 mL/kg/min) and 6 weeks (+1.9 ± 1.8 mL/kg/min) of HIIT (both *p* < 0.001), with 6-week HIIT required to elicit improvements in VO_2peak_ (+3.0 ± 6 mL/kg/min; *p* = 0.04). Exercise tolerance increased after 2 (+15 ± 15 W), 4 (+17 ±11 W), and 6 weeks (+16 ± 11 W) of HIIT (all *p* < 0.001), with no difference in increase between the groups.	Compliance: 100% training compliance reported AEs: None reported
Herrod et al. (2020b) [27]	Frequency: 3×/week for 6 weeks Intervals: 5 intervals at 90–110% of PPO for 1 min Rest: 90 s of active recovery Modality: Cycle ergometer	For SBP, there was a main effect of time (P<0.001) and a significant group x time interaction (P= 0.04), with significant reductions in both the HIIT (142(15) vs. 133(11); −9(9) mmHg, P<0.001) and IHG (139(15) vs. 130(12); −9(9)mmHg, P= 0.002) groups. ☐ere was no significant change in either the RIPC (138(15) vs. 134(14); −4(5), P= 0.17) or control (130(10) vs. 128(10); −1(6), P= 0.96) groups Systolic blood pressure significantly decreased in the HIIT (− 9 ± 9 mmHg) and isometric handgrip training groups (− 9 ± 9 mmHg). There was no significant change in the control or remote ischemic preconditioning groups.	Mean (SD) training compliance was 99(3)%, and there were no adverse events Mean (SD) training compliance was 99(3)%, and there were no adverse events Compliance: Mean (SD) was 99(3) % AEs: None reported
Hwang et al. (2016) [28]	Frequency: 4×/week for 8 weeks Intervals: 4 intervals at 90% HR_peak_ for 4 min Rest: 3 min at 70% HR_peak_ Modality: Non-weight-bearing all-extremity ergometer	Primary outcome—see feasibility and tolerability. Secondary outcomes—VO_2peak_ improved by 11% and ejection fraction improved by 4% in HIIT, no change was seen in MCT or control. Insulin resistance decreased by 26% in the HIIT group only. Diastolic function, body composition, lipid, and glucose did not change.	Dropouts: Of 51 participants randomized, 16% did not complete the study (control: 2 unable to contact; MCT: 2 lack of motivation, 1 family conflict, 1 schedule conflict; HIIT: 1 family conflict, 1 schedule conflict) Compliance: rate by participants was 88% AEs: None in HIIT HIIT was deemed to be feasible
Kim et al. (2017) [29]	Frequency: 4×/week for 8 weeks Intervals: 4 intervals at 90% HR_peak_ for 4 min Rest: 3 min active recovery at 70% HR_peak_ Modality: All-extremity non-weight-bearing ergometer	Arterial stiffness improved after MCT only (decrease in carotid to femoral pulse wave velocity and increase in common carotid artery compliance). No change was seen in HIIT.	Dropouts: 9 of 49 subjects did not complete intervention (HIIT: family issues, schedule conflict; MCT: lack of motivation, inability to contact for follow-up. 2 subjects were excluded from analysis (1 in MCT due to unrelated illness, 1 in control group due to non-compliance). Compliance: Similar between MCT and HIIT (both 90%). AEs: None reported Exercise training was described as "well-tolerated.”
Kovacevic et al. (2020) [30]	Frequency: 3×/week for 12 weeks Intervals: 4 intervals at 90–95% HR_peak_ for 4 min Rest: 3 min active recovery at 50–70% HR_peak_ Modality: Treadmill	HIIT and MCT interventions induced similar cardiorespiratory fitness adaptations from pre- to post-test, such that exercise training led to the greatest increases in predicted VO_2peak_. High-interference memory significantly improved following HIIT but not MCT or control. There was no main effect of group on brain-derived neurotrophic factor	Dropouts: 13 participants withdrew during training (HIIT = 3, MCT = 5, stretching = 5) Compliance: All participants completed at least half of training protocol.
Krusnauskas et al. (2018) [31]	3 SIT protocols: 1) 6 intervals of 5 s at “all-out” intensity (90-s rest), 2) 3 intervals of 30 s at “all-out” intensity (4-min rest), or 3) 3 intervals of 60 s at submaximal intensity (4-min rest) Frequency: Single session Modality: Cycle ergometer	Decrease in torque ratio represented low-frequency fatigue and was more evident in the 30 s and 60 s protocols. In young women, low volume (6 × 5 s) exercise induces physiological stress and is effective. In older women, longer intervals (3 × 60 s) are more stressful than shorter but still tolerable.	Acceptance of protocol: 6 × 5 s cycling was the most preferred method in both age groups. Perceived enjoyment was similar in both groups.
Linares et al. (2020) [32]	Frequency: Single session Intervals: 10 intervals at 90% PPO for 1 min Rest: 1 min at 10% PPO Modality: Cycle ergometer	There were similar peaks in oxygen consumption (in women alone) and in HR (both women and men) when comparing HIIT and maximal exercise. There was a greater cardiopulmonary response to HIIT compared with MCT. When PPO was used for exercise prescription there was considerable individual variability in work intensity seen.	Not given
McSween et al. (2020) [33]	Frequency: Single session Intervals: 4 intervals at 85–95% HR_peak_ for 4 min Rest: 3 min at 50-65% HR_peak_ Modality: Cycle ergometer	In lower baseline learning performers, the MCT group performed significantly better at the immediate recall task when compared with the stretching group, whereas this difference was not observed between the stretching and HIIT group and the MCT and HIIT group.	Not given
Mejias-Pena et al. (2016) [34]	Frequency: 2×/week for 8 weeks Intervals: 0–4 intervals (progressive) at 90–95% HR_max_ for 1 min Rest: 70-75% HR_max_ for 4–7 min Modality: Cycle ergometer	Less loss of autophagic activity was seen in older adults after HIIT. Peak oxygen uptake increased post-intervention in the training group.	Not given
Mekari et al. (2020) [35]	Frequency: 3×/week for 6 weeks Intervals: 15-s intervals at 100% PPO Rest: 15-s passive recovery at 0% PPO Modality: Cycle ergometer	VO_2max_ significantly improved in all groups following training, but HIIT and MCT improved more than RT. The HIIT group had the greatest improvement in VO_2max_. Regarding cognitive flexibility, the HIIT group exhibited a faster reaction time (from 1250 ± 50 to 1100 ± 50 ms; *p* < 0.001) in switching.	Not given
Nakajima et al. (2010) [36]	Frequency: 2×/week for 6 months Intervals: 3 min at > 70% peak aerobic capacity Rest: 3 min at 40% peak aerobic capacity Time: > 26 min Modality: Walking	Methylation of *ASC* gene (inflammatory mediator involved in initiating innate immunity) decreased significantly with age (young control vs. older control, p<0.01), which is indicative of an age-dependent increase in ASC expression. Compared to the older control group, the degree of ASC methylation was higher in the older HIIT group.	Not given
Nederveen et al. (2015) [37]	Frequency: Single session Intervals: 10 intervals at 90-95% VO_2max_ for 1 min Rest: “low-intensity” for 1 min Modality: Cycle ergometer	Satellite cell response to exercise: Specific to type 1 fibers, expansion occurred 24 and 48 h post-treatment in the HIIT group. HIIT and RT groups showed greater response 24-h post-treatment than MCT. HIIT was nearly as effective as RT in increasing the number of active satellite cells following an acute bout of exercise.	Not given
O’Brien et al. (2020) [38]	Frequency: 3×/week for 6 weeks Intervals: 15-s intervals at 100% PPO Rest: 15-s passive recovery at 0% PPO Modality: Cycle ergometer	Resting HR decreased in the MCT group only. HIIT group had lower systolic, diastolic, and mean arterial blood pressures post-training but MCT only decreased diastolic blood pressure. RT did not change any systemic resting hemodynamic measurements. Resting brachial artery blood flow and vascular conductance (both, *p* < 0.003) were greater after HIIT only. The HIIT and MCT similarly increased brachial artery flow-mediated dilation (pre–post both, *p* < 0.001), but only HIIT improved brachial artery low flow-mediated constriction.	Not given
Osuka et al. (2017) [39]	Frequency: Single session Intervals: 3 intervals at 75–85% VO_2peak_ for 2–3 min Rest: 1-2 min at 50% VO_2peak_ Modality: Cycle ergometer	Primary—feasibility. Secondary—exercise intensity achieved: %VO_2peak_ achieved during the interventions was greater in HIIT than MCT, %HR_peak_ achieved during exercises were different between protocols throughout, including their peak HR reached. The ratings of perceived exertion were similar between groups.	Compliance: Completion rates were similar between treatments (MCT: 95.2%, HIIT: 100%) AEs: No severe AEs reported. During HIIT cool-down, one participant had transient asymptomatic tachycardia for less than 1 min.
Stockwell et al. (2012) [40]	Frequency: Single session Intervals: 10 intervals at 70% VO_2max_ for 1 min Rest: 30% VO_2max_ for 1 min Modality: Cycle ergometer	Greater HR changes were seen in HIIT compared to MCT. Compared to MCT, VO_2_ was 16% higher during HIIT though this was not statistically significant. Similar ratings of perceived exertion were seen for both protocols.	Dropouts: None Acceptance of protocol: Self-report suggested that enjoyability of HIIT was higher than MCT and may contribute to increased adherence.
Storen et al. (2017) [41]	Frequency: 3×/week for 8 weeks Intervals: 4 intervals at 90–95% HR_max_ for 4 min Rest: 3 min at 70% HR_max_ Modality: Treadmill and cycling	After HIIT, all age cohorts significantly increased VO_2max_ by 9–13%. These changes did not differ between age cohorts. No change was seen in HR_max_.	Compliance: Participants were only included in results if compliance was > 80% and if their initial VO_2max_ was representative of their age group. Mean compliance was reported as 92% +- 4% (no significant difference between age groups or gender) AEs: None reported
Venckunas et al. (2019) [42]	3 SIT protocols: 1) 6 intervals of 5 s at “all-out” intensity (90-s rest), 2) 3 intervals of 30 s at “all-out” intensity (4-min rest), or 3) 3 intervals of 60 s at submaximal intensity (4-min rest) Frequency: Single session Modality: Cycle ergometer	All protocols increased the blood lactate concentration and decreased maximal voluntary contraction and electrically stimulated knee extension in young and especially untrained young men. The higher-volume sessions more markedly suppressed contractile function and also increased serum testosterone in untrained groups.	Not given
Vogel et al. (2011) [43]	Frequency: 2×/week for 9 weeks Intervals: 6 intervals at 90% Max tolerated power for 1 min Rest: VT_1_ for 4 min Modality: Cycle ergometer	Significant improvement of maximum tolerated power, VO_2peak_ and maximal minute ventilation was seen for both age groups compared to baseline. In the “older senior” group post-HIIT, some measures of cardio-respiratory response were not statistically different from the “young senior” responses pre-HIIT (in women: maximum tolerated power; in men and women: VO_2peak_, maximal minute ventilation, first ventilatory threshold).	Compliance: 100% adherence rate to training program AEs: No training-related AEs reported
Windsor et al. (2018) [44]	Frequency: Single session Intervals: 12 intervals at 70% PPO for 1 min Rest: 1 min at 10% PPO Modality: Cycle ergometer	Plasma cytokine concentrations: IL6 & 10 increased in both groups immediately post either HIIT or MCT; no difference between exercise and non-exercisers; no changes in TNF-a.	Not given
Wyckelsma et al. (2017) [45]	Frequency: 3×/week for 12 weeks Intervals: 4 intervals at 90–95% HR_peak_ for 4 min Rest: 4 min at 50–60% HR_peak_ Modality: Cycle ergometer	HIIT increased VO_2peak_ by 16% and increased the peak work rate by 11% with no significant reduction in the rise of [K+]. Muscle Na+,K+-ATPase NKA content increased by 11% in the HIIT group with no change in control group.	Dropouts: 1 due to ill health unrelated to study, one due to high blood pressure after exercise. Compliance: Not including dropouts, all completed at least 83% of sessions AEs: 5 participants had mild vasovagal episodes during training without further incidents
Yasar et al. (2019) [41]	Frequency: 2 sessions separated by 3 or 5 days of recovery Intervals: 3 intervals of 20 s “all-out” intensity Rest: 3 min self-paced Modality: Cycle ergometer	A large effect of age was seen on PPO, with the older group having a lower PPO. Both groups could recover in 3 or 5 days.	Not given
Yoo et al. (2017) [46]	Frequency: Single session Intervals: 4 intervals at 90% HR_peak_ for 4 min Rest: 3 min at 70% HR_peak_ Modality: Treadmill	In men, FMD was similarly attenuated by 45% after HIIT and by 37% after MCT. In women, FMD did not significantly change after HIIT or MCT.	Not given

*HIIT* high-intensity interval training; *SIT* sprint interval training; *MCT* moderate-intensity continuous training; *RT* resistance training; *MAP* mean arterial pressure; *HDL *high-density lipoprotein; *LDL* low-density lipoprotein; *FMD* flow-mediated dilation; *PPO* peak power output; *HR* heart rate; *VO*_*2*_volume of oxygen consumption; *AE* adverse events

**Table 3:** HIIT studies in cardiovascular disease

Article	HIIT/SIT protocol	Outcomes	Feasibility/tolerability
Bailey et al. (2018) [47]	Frequency: Single session Intervals: 12 intervals at 70% PPO for 1 min Rest: 1-min recovery at 10% PPO Modality: Cycle ergometer	Brachial artery FMD increased after MCT and decreased after HIIT in both AAA and healthy cohorts.	Not given
Currie et al. (2012) [48]	Frequency: Single session Intervals: 10 intervals at 80% PPO for 1 min Rest: 10% PPO for 1 min Modality: Cycle ergometer	Mean HR and total work performed were higher in MCT compared to HIIT. In spite of this, there was no significant difference between the two interventions in the increase of brachial artery FMD 60 min post-exercise (absolute or relative values).	Compliance: All completed HIIT; 2 could not complete MCT due to volitional fatigue.
Currie et al. (2013) [49]	Frequency: 2×/week for 12 weeks Intervals: 10 intervals at 89% PPO for 1 min Rest: recovery at 10% PPO for 1 min Modality: Cycle ergometer	Relative increase in FMD and improved VO_2peak_ post-training in both groups with no significant difference between groups.	Dropouts: 4 (not included in results): 3 had changes to beta-blockers and one was put on calcium channel blocker. AEs: None in either group
dos Santos et al. (2018) [50]	Frequency: Single session Intervals: 4 intervals at 85–90% HRR for 4 min Rest: 2-min active recovery at 50% HRR Modality: Cycle ergometer	The HIIT session promoted a greater systolic hypotensive effect compared to the MCT session. There was no significant difference in post-exercise diastolic hypotension between groups.	Dropouts: Of 39 participants recruited, 20 did not attend one of the exercise sessions (not mentioned which one), 4 did not reach target zone for the exercise and were excluded. Acceptance of protocol: Participants subjectively reported that their comfort level was higher in HIIT than in MCT.
Guiraud et al. (2009) [51]	4 SIT protocols: A) 15-s intervals (15-s passive rest: 0% MAP) B) 15-s intervals (15-s active rest: 50% MAP) C) 60-s intervals (60-s passive rest: 0% MAP) D) 60-s intervals (60-s active rest: 50% MAP) Frequency: Single session Intervals: 100% MAP Time: As long as tolerated or 35 min maximum Modality: Cycle ergometer	All protocols had similar time spent above 80% VO_2peak_. Protocol A had a significantly lower rating of perceived exertion at the end of the session. Significantly, 63% of participants were able to complete the entire duration of this exercise (compared to 16%, 42%, and 0% of protocols B, C, and D, respectively. 18 out of 19 participants rated protocol A as both the preferred protocol.	Dropouts: 1 due to injury due to recreational activity and not included in results AEs: 2 patients had vagal episodes after one HIIT protocol. 3 subjects presented myocardial ischemia and developed mild angina during the SIT exercises. Maximal ST-depression never exceeded 2mm. Symptoms and ST-depression resolved during passive recovery.
Helgerud et al. (2009) [52]	8-weeks of plantar flexion HIIT (alternating legs, 4-min intervals at 80%Wmax) followed by 8 weeks of treadmill HIIT: Frequency: 3×/week for 8 weeks Intervals: 4 intervals at 90–95% HR_peak_ for 4 min Rest: 3-min active rest for recovery Modality: Treadmill	Aerobic capacity and CV function were improved after plantar flexion training (plantar flexion VO_2peak_ increased 14.8%, treadmill VO_2peak_ increased 16.8%, time to exhaustion increased 58.5%, PPO increased 61.4%). These changes were increased by further treadmill training (additional treadmill VO_2peak_ increase by 9.9%, time to exhaustion increased 16.1% and Q and SV increased by 33.4% and 25.1%, respectively).	Compliance: Participants excluded from results if they did not attend at least 85% of training sessions.
Moore et al. (2020) [53]	Frequency: ≤ 40 sessions in 10 weeks Interval: ≤ 40 min targeting 70–85% HR_max_ Rest: Breaks as needed Modality: Stepping	Average steps per day in HCT (5777 ± 2784) were significantly greater than during usual care (3917 ± 2656; *p* < 0.001). Statistically different and clinically meaningful changes in self-selected speed (0.39 ± 0.28 versus 0.16 ± 0.26 m/s) and fastest gait speed (0.47 ± 0.41 versus 0.17 ± 0.38 m/s; both *p* < 0.001) were observed following HCT vs usual care. Intensity achieved: HCT participants on average maintained the target intensities for over 30% of each session.	AEs: Falls outside of therapy were most commonly reported (9 in control and 11 in HCT). During usual care only there was a report of infection and 7 transfers to acute care for medical issues, syncope, and unknown reasons.
Nepveu et al. (2017) [54]	Frequency: Single session Intervals: 3 intervals at 100% Wpeak for 3 min Rest: recovery at 25% Wpeak for 2 min Time: 15 min Modality: Recumbent stepper	Motor task skill learning and retention was higher in HIIT group (9% improvement vs 4% decay in control). A maximal graded exercise test did not result in significant changes in corticospinal excitability.	Dropouts: Out of 22 participants included in the study: data were lacking for 1 retention test and for 2 transcranial magnetic stimulation tests.
Reichert et al. (2016) [55]	Frequency: 2×/week for 28 weeks Intervals: 6–12 intervals at Borg scale 15–18 for 2–4 min Rest: recovery for 0.5–1 min Modality: Deep water running	Similar and significant improvement seen in both groups post-exercise in measures of functional fitness: foot up-and-go (12% in both groups), flexibility of lower limbs and strength in upper and lower limbs (number of repetitions improved by over 40% in both groups), and 6 min walk test (12% in HIT group, 4% in MCT group). Both systolic and diastolic BP was significantly decreased in both groups post-training. This change was similar between both groups for systolic pressure but greater in diastolic pressure after continuous training.	Dropouts: HCT: 1 allergy, 1 surgery, 2 discontinued, 1 refused to participate in the assessments; from HIIT: 2 excessive absence, 3 abandoned the study. Compliance: Samples that did not obtain at least 80% frequency in the sessions were excluded.
Sosner et al. (2016) [56]	Frequency: Single session Intervals: 15 s at 100% PPO Rest: 15-s passive recovery Time: 2× 10-min sets Modality: Cycle ergometer (on dryland or immersed in water)	Similar decrease in systolic BP was seen in all groups 4 h after exercise. 24-h ambulatory BP was significantly decreased post-exercise only in HIIT groups, with increased change seen in immersed (compared to dryland) protocol.	Not given
Tew et al. (2017) [57]	Frequency: 3×/week for 4 weeks Intervals: 8 or 4 intervals at the Rate of Perceived exertion for legs: 5, or the Rate of Perceived exertion for chest/breathlessness: 7 for 2 or 4 min Rest: active recovery for 2 min Modality: Cycle ergometer	Primary—see feasibility and tolerability. Secondary—No significant change in cardio-respiratory fitness was seen between groups. Difference in post-op morbidity, mortality, and quality of life between groups was trivial to small.	Dropouts: Rate of screening: 100%; Eligibility of participants: 43.2%; Recruitment: 22.1%; Retention: 91%; Outcome completion: 79–92% Compliance: Overall attendance: 75.8%. Exercise intensity was generally lower than what had been intended. AEs: One participant experienced prodromal symptoms on 4 occasions when power output increased over 80 W. Symptoms resolved by decreasing workload. Acceptance of protocol: The program was scored as “enjoyable.”
Windsor et al. (2018) [58]	Frequency: Single session Intervals: 12 intervals at 70% PPO for 1 min Rest: 1-min recovery at 10% PPO Modality: Cycle ergometer	Healthy subjects had higher mean power outputs in both MCT and HIIT groups. Greater anti-inflammatory response was seen in HIIT compared to MCT groups. This was further augmented by AAA. Post-MCT, there was a modest and transient increase in IL-6 and MMP-9 in healthy and AAA patients. 90 min post-HIIT, there was a decrease in MMP-9 in both populations and lower TNF-α in AAA group.	Not given

*HIIT* high-intensity interval training; *SIT* sprint interval training; *HCT *high-intensity continuous training; *MCT* moderate-intensity continuous training; *PPO* peak power output; *W*work; *FMD* flow-mediated dilation; *CV* cardiovascular; *AAA* abdominal aortic aneurysm; *HR* heart rate; *MAP* max aerobic power; *VO*_*2*_ volume of oxygen consumption

**Table 4:** HIIT studies in cardiac disease

Article	HIIT/SIT protocol	Outcomes	Feasibility/tolerability
Angadi et al. (2015) [59]	Frequency: 3×/week for 4weeks Intervals: 4 intervals at 85–90% HR_peak_ for 4 min Rest: Active recovery for 3 min Modality: Treadmill	Diastolic BP was reduced after HIIT only. VO_2peak_ increased by 9% post-HIIT but not post-MCT. Ventilation threshold, HR_peak_, respiratory exchange ratio was unchanged in both groups. Brachial artery FMD was unchanged post-intervention in both groups. Diastolic dysfunction was reduced after HIIT by approximately 1 grade.	Dropouts: 4 participants excluded (2—noncompliance with baseline teste procedures, 1—change in employment status, 1—noncardiovascualr illness. Compliance: 13 subjects completed 100% of sessions, 2 subjects completed 11 of 12 sessions. AEs: No reported musculoskeletal injuries and no significant cardiac events reported.
Ellingsen et al. (2017) [60]	Frequency: 3×/week for 12 weeks Intervals: 4 intervals at 90–95% HR_max_ for 4 min Rest: 3-min active recovery at moderate intensity Modality: Treadmill or cycle ergometer	At 12 weeks post-baseline, both changes in left ventricular end-diastolic diameter and in VO_2peak_ were similar between HIIT and MCT groups but larger than the control group. No difference was seen in LVEF or in respiratory quotient between groups. No differences in endpoints were seen between groups at 52 weeks.	After initiating the training program Dropouts: 9 participants dropped out due to serious adverse events, 7 withdrew or were lost to follow-up. Compliance: Adherence to supervised training ranged from 34-36 of 36 sessions. AEs: No statistically significant difference of serious AEs between groups but the HIIT group had more cardiovascular AEs during the intervention period and for the remainder of the year.
Fu et al. (2013) [61]	Frequency: 3×/week for 12 weeks Intervals: 5 intervals at 80% VO_2peak_ for 3 min Rest: 3 min at 40% VO_2peak_ Modality: Cycle ergometer	Aerobic fitness was significantly increased in HIIT group only (increase in ventilatory efficiency and cardiac-cerebral-muscular hemodynamic response to exercise).	Dropouts: HIIT—1, MCT—2, control—2; Results of participants who dropped out were included in pre-intervention data.
Iellamo et al. (2014) [62]	Frequency: 3×/week for 12weeks Intervals: 4 intervals at 75–80% HRR for 4 min Rest: Recovery at 45–50% HRR for 3 min Modality: “Uphill” treadmill walking	Ambulatory blood pressure did not significantly change but trended towards decreasing in both groups. Daytime diastolic BP was reduced significantly in HIIT compared to MCT. No significant change in LVEF or LVDD see in either group compared to baseline. VO_2peak_ increased significantly and similarly in both groups. Both groups showed significant and similar decrease in fasting glycaemia, insulin and homeostatic model assessment-IR except the HOMA-IR was further reduced in HIIT than MIT.	Dropouts: 1 participant in HIIT group and 2 in MCT group discontinued study to due unwillingness to continue in study. Compliance: HIIT group average = 31.6/36 sessions; MCT group average = 30.1/36 sessions AEs: None reported
Isaksen et al. (2015) [63]	Frequency: 3×/week for 12 weeks Intervals: 4 intervals at 85% HR_max_ for 4 min Rest: Recovery at 60–70% HR_max_ for 3 min Modality: Cycle ergometer /treadmill	Significant increase in VO_2_ uptake (5.7% increase in HIIT vs 4.1 decrease in control), cycle ergometer workload, and endothelial function was seen in HIIT compared to control. See feasibility and safety as further outcomes.	Dropouts: 35 of 38 recruited completed the study: one from control group, two from HIIT due to medical complications: repeated haematuria following exercises and diagnosed with urothelial carcinoma and device-related infection. Compliance: Average attendance rate was 98% with none completing less than 75% of the planned sessions and 20 completing 100% AEs: None reported, including symptomatic arrhythmias, sustained arrhythmias, antitachycardia pacing, or implantable cardioverter defibrillator discharge.
Isaksen et al. (2016) [64]	Frequency: 3×/week for 12 weeks Intervals: 4 intervals at 85% HR_max_ for 4 min Rest: Active recovery for 3 min Modality: Cycle ergometer /treadmill	In HIIT only, significant increase in VO_2peak_ was seen. Some improvements in anxiety and depression scores (SF-36 and HADS-D) were seen in HIIT group at 12 weeks. At 2-year follow-up, the HIIT group had maintained scores, or scores trended towards baseline values. This was significantly improved over controls who had no change at 12 weeks and had deteriorated scores at 2-year follow-up. At 2-year follow-up, the control group reported significantly more time spent sitting during the day compared to the HIIT group. Non-significantly, the HIIT group also had more physical activity per week. No significant differences between groups regarding hospitalization and implantable cardioverter defibrillator shocks at 2-year follow-up.	Dropouts: 1, not in results Compliance: 26 completed 2-year follow-up assessment. Mean attendance rate = 97.5%. Mean reported Borg score was 15.2 during intervals.
Munch et al. (2018) [65]	Frequency: 3×/week for 6 weeks Intervals: 8 intervals at 90% 1-leg Wmax for 4 min (alternating legs) Rest: 1.5–2-min recovery Modality: Cycle ergometer (1-legged)	In both HF and healthy populations, HIIT increased aerobic capacity and improved ability to override sympathetic vasoconstriction (arterial infusion of tyramine) during exercise. The peak vasodilatory responsiveness to ATP infusion was less in the HF population. Acetylcholine-induced vasodilation in the HF population was increased after HIIT.	Not given
Spee et al. (2020) [66]	Frequency: 3×/week for 3 months Intervals: 4 intervals at 85-95% VO_2peak_ for 4 min Rest: 3 min active rest Modality: Cycle ergometer	After cardiac resynchronization therapy (both groups), VO_2peak_ increased (17 ± 5.3 to 18.7 ± 6.2 ml/kg/min, p < 0.05). After HIIT there was a non-significant increase of 1.4 ml/kg/min (*p* = 0.12). Peak cardiac output did not change significantly after cardiac resynchronization therapy or HIIT. LVEF increased 25% after resynchronization therapy but not after HIIT.	Dropouts: After randomization, two participants could not complete the protocol due to orthopedic complaints.
Thijssen et al. (2019) [67]	Frequency: 2×/week for 12 weeks Intervals: 10 intervals at 90% Wmax for 1 min Rest: 30% Wmax for 2.5 min Modality: Cycle ergometer	VO_2peak_ (as percentage of predicted VO_2peak_) and maximum workload increased after training with no difference seen between training groups. No significant change in FMD, cardiac function, or health-related quality of life (SF-36 total score) was seen.	Dropouts: 4 dropouts after allocation (2 due to musculoskeletal complaints and 2 due to progression of HF—one of each in each group) Compliance: 100% as missed sessions were rescheduled

*HIIT* high-intensity interval training; *MCT* moderate-intensity continuous training; *HR* heart rate; *VO*_*2*_ volume of oxygen consumption; *BP* blood pressure; *FMD* flow-mediated dilation; *LVEF* left-ventricular ejection fraction; *HF* heart failure; *AE* adverse events

**Table 5:** HIIT studies in metabolic disease

Article	HIIT/SIT protocol	Outcomes	Feasibility/tolerability
Andonian et al. (2018) [68]	Frequency: 3×/week for 10 weeks Intervals: 10 intervals at 80–90% HRR for 1–1.5 min Rest: Recovery at 50–60% HRR for 1–1.5 min Modality: Treadmill (graded)	Muscle remodeling markers (plasma galectin-3, skeletal muscle cytokines, muscle myostatin concentrations) were unchanged from baseline after training in both populations. Both groups had an increase in VO_2peak_ and Disease Activity Score-28 after training.	Not given
Bartlett et al. (2020) [69]	Frequency: 3×/week for 10 weeks Intervals: 60–90 s at 80–90% VO_2_ reserve Rest: 60–90 s at 50–60% VO_2_ reserve Time: 30 min Modality: Treadmill walking	In both groups after training there was significant decrease in fasting glucose and insulin and improved glucose control and insulin sensitivity (all p < 0.05). Before training, VO_2peak_ in the older group was significantly less than that of the younger group (p < 0.001) but increased by 16 ± 11% following training (*p* = 0.002), decreasing the difference by 6%.	Not given
Boukabous et al. (2019) [70]	Frequency: 3×/week for 8 weeks Intervals: 6 intervals at 90% HRR for 1 min Rest: 2-4 min recovery at 40% HRR Modality: Treadmill	Neither exercise group resulted in a change in body composition. Total cholesterol, non-HDL cholesterol, and Framingham risk decreased similarly in both groups. Physical capacity (6-min walk test) significantly increased in both groups while maximal strength and VO_2peak_ were unchanged.	Dropouts: None Compliance: No difference in completion rate between groups (HIIT: 92.7%, MCT 94.7%).Acceptance of protocol: Affective response before and after each session was high and similar between HIIT and MCT and was stable throughout intervention.
Hwang et al. (2019) [71]	Frequency: 4×/week for 8 weeks Intervals: 4 intervals at 90% HR_peak_ for 4 min Rest: 3 min at 70% HR_peak_ Modality: All-extremity non-weight-bearing ergometer	Primary—feasibility and tolerability; Secondary—aerobic fitness increased significantly and similarly in both groups (VO_2peak_ increased by 10% in HIIT and 8% in MCT; Maximal exercise test duration increased by 1.8 min in HIIT and 1.3 min in MCT; VT increased by 11% in HIIT and 14% in MCT). Percent body fat decreased by 1% in MCT, increased by 0.9% in control, and was unchanged in HIIT. Glycemic control and lipids were unchanged by interventions	Dropouts: 8 withdrew and were not included in results. 22% participants in HIIT and 16% in MCT (HIIT: schedule conflicts, prior medical issues, plantar fasciitis; MCT: ergometer seat and hurricane). Compliance: Similar attendance in both groups and one in each group missed a session due to exercise-related fatigue. AEs: HIIT: 1 participant experienced dyspnea, one participant on insulin had dizziness and hypotension once following HIIT, neither experienced hypoglycemia. Both recovered with rest and rehydration. During initial sessions, some found ergometer seat to be uncomfortable and one withdrew. No serious AEs were noted requiring hospitalization or medical treatment. Acceptance of protocol: Most participants reported the interventions to be enjoyable except for a few in MCT who complained of boredom.
Karstoft et al. (2017) [72]	Frequency: 10 sessions in 2 weeks Intervals: 10 intervals at 89% VO_2peak_ for 3 min Rest: 54% VO_2peak_ for 3 min Modality: Treadmill	Neither intervention had an impact on the resting metabolic rate and mean oxygen consumption and heart rates were similar between the treatment groups. Neither intervention resulted in changes in physical fitness or body composition. Measures of glycemic control, however, were seen to be improved in the HIIT group but not MCT or control.	Compliance: 99% adherence in both MCT and HIIT.
Maillard et al. (2016) [73]	Frequency: 2×/week for 16 weeks Intervals: Maximum 60× 8-s intervals at 80% HR_max_ Rest: 12-s active recovery Time: 20 min Modality: Cycle ergometer	Both HIIT and MCT resulted in similar decrease in whole-body fat mass (HIIT: − 2.5% ± 1.3%; MCT: − 3.2% ± 1.2%). HIIT resulted in a significantly larger decrease in total abdominal and visceral fat mass. HbA_1c_ and triglyceride-to-HDL ratio decreased after interventions in both groups. After 16 weeks, levels of physical activity scores, total energy intake, and macronutrient consumption did not change in either group.	Dropouts: 1 from MCT group for personal reasons.
Mohammadi et al. (2017) [74]	Frequency: 3×/week for 8 weeks Intervals: 4–8 intervals at 90% HRR for 4 min Rest: Active recovery for 2 min Modality: Not given	Serum level of adipokines (chemerin and visfatin) decreased significantly after 8 weeks HIIT. Weight, BMI, and percentage of body fat all decreased significantly after HIIT intervention.	Not given
Pandey et al. (2017) [75]	Frequency: 3×/day, 5days/week for 12 weeks Intervals: 85% HR_max_ for 10 min Rest: At least 2h between sessions Modality: Variable, both supervised (2×/week) and at home (3×/week)	Cardiometabolic measures were significantly improved in HCT compared to MCT: Specifically, a significant decrease in BMI, reduction in HbA_1c_ and greater decrease in LDL (HIIT: − 11% vs. MCT: − 4%) and greater increase in HDL (HIIT: 22% vs. MCT: 3%).	Compliance: Adherence poor in both groups but better in the HIIT group. Exercise goal was 600 min of exercise/month in both groups. Compliance ranged from about 200 min to nearly 600 min/month. (60.3% for MCT, and 76.7% for HIIT)

*HIIT*: high-intensity interval training; *MCT*: moderate-intensity continuous training; *VO*_*2*_: volume of oxygen consumption; *HDL*: high-density lipoprotein; *LDL*: low-density lipoprotein; *BMI*: body mass index; *HbA1c*: glycosylated hemoglobin; *HRR*: heart rate reserve; *HR*: heart rate; *VT*: ventilatory threshold; *AE* = adverse events

**Table 6:** HIIT studies in other clinical populations

Article	HIIT/SIT protocol	Outcomes	Feasibility/tolerability
Banerjee et al. (2018) [76]	Frequency: 2×/week for 3–6 weeks Intervals: 6 intervals at 70–85% HR_max_ for 5 min Rest: recovery 2.5-min active rest Modality: Cycle ergometer	Primary—Feasibility and tolerability. Secondary—Improvements in peak values of oxygen pulse, minute ventilation, and power outage in exercise group vs controls.	Dropouts: Of 112 eligible patients, recruitment = 53.5% (60), attrition = 8.3%. 5 of the 60 recruited patients dropped out of the study (2 unfit for surgery following randomization and 3 opted for radiotherapy after follow-up endurance test). Compliance: Median number of exercise sessions attended = 8 (range 1–10) in 3–6 weeks. 4 did not meet max rating of perceived exertion score ≤ 16. AEs: None reported.
Devin et al. (2019) [77]	Frequency: 3×/week for 4 weeks Intervals: 4 intervals at 85–95% HR_max_ for 4 min Rest: 3-min recovery period Modality: Cycle ergometer	Primary: Cancer cell number after incubation with patient serum (cells in serum immediately post HIIT sig decreased; no change from serum at 120 min post-exercise) Secondary: cell apoptosis (no difference/change), systemic marker analyses (immediately post HIIT—increase TNF-a, IL-6/8, insulin; all returned to baseline at 120 min except insulin).	Not given
Fiorelli et al. (2019) [78]	Frequency: Single session Intervals: 7 intervals between Borg scale 13–17 for 1 min Rest: recovery at 9–11 on Borg scale for 2 min Modality: Cycle ergometer	Both MCT and HIIT improved immediate auditory memory but HIIT also improved attention and sustained attention. Working memory was not impacted by either intervention.	Authors state that both exercise interventions were well-tolerated.
Hoffmann et al. (2016) [79]	Frequency: 3×/week for 16 weeks Intervals: 3 intervals at 70–80% HR_max_ for 10 min Rest: recovery of 2–5-min rest Modality: Various	Per intention-to-treat analysis, HIIT did not show any change from baseline in cognitive scores, quality of life, or activities of daily living. The intervention group did show a greater change towards less severe neuropsychiatric symptoms. In subjects who adhered to the intervention, the Symbol Digit Modalities Test score significantly improved compared to control.	Compliance: 76% of the HIIT group attended more than 80% of HIIT sessions, 78% of HIIT participants exercised at intensity over 70% of HR_max_. 62% of the HIIT group did both above criteria. AEs: In the HIIT group, 35 AEs and 7 serious AEs were reported. Of those suspected to be related to the intervention, 6 were MSK problems, 6 were dizziness or faintness, and 1 possibly related was atrial fibrillation.
Keogh et al. (2018) [80]	Frequency: 4×/week for 8 weeks Intervals: 5 intervals at 100rpm at a level at which it is “quite difficult to complete sentences” for 45s Rest: recovery at 70 rpm for 90 s Modality: Home cycle ergometer	Primary—Feasibility and safety. Secondary—Both HIIT and MCT similarly and significantly improved their health-related quality of life (measured by WOMAC scores) but HIIT also significantly improved physical performance as measured by the Timed Up and Go test (which was also significantly greater than the MCT group) and the 30 s Sit-to-Stand test. There was no change in body composition, gait speed, or Lequense index in either group.	Dropouts: Of 27 initially enrolled, 17 participants completed the study (dropout rate of 37%). Compliance: Adherence high (MCT = 88%, HIIT = 94%). AEs: 3 individuals reported AEs (1 MCT, 2 HIIT). Total of 28 AEs, 24 of these by 1 HIIT participant.
Mitropoulos et al. (2018) [81]	Frequency: 2×/week for 12 weeks Intervals: 100% PPO for 30 s Rest: 30-s passive recovery Time: 30 min Modality: Cycle ergometer or arm crank	Primary: Peak oxygen uptake increased similarly and significantly in both exercise groups compared to baseline. The arm-crank group had improved cutaneous vascular conductance compared to baseline after intervention. Both exercise groups had increased life satisfaction scores and decreased discomfort and pain of Raynaud’s phenomenon post-intervention compared to control.	Dropouts: 1 in each exercise group. Compliance: Compliance in the cycling group was 88% compared to 92% in the arm-crank group. AEs: No exercise-related complications were reported. Acceptance of protocol: Enjoyment scores for both exercise groups were high, averaging “good.”
Northey et al. (2019) [82]	Frequency: 3×/week for 12 weeks Intervals: 4–7 intervals over 90% HR_max_ by 4th interval for 30 s Rest: 2 min active recovery Time: 20–30 min Modality: Cycle ergometer	HIIT had moderate to large positive effects compared to MCT and control on cognitive performance including episodic memory, working memory, executive function, cerebral blood flow, and cerebrovascular reactivity, but these were not statistically significant. HIIT also significantly increased VO_2peak_ by 19.3% while MCT had non-significant increase of 5.6% and control had a decrease of 2.6%.	Dropouts: None Compliance: Adherence similar between HIIT and MCT (78.7 % attendance in HIIT) AEs: None reported
Rizk et al. (2015) [83]	Acute bout followed by 12-week training intervention Frequency: 3×/week for 12 weeks Interval: 30-s intervals at 100% Wpeak Rest: 30-s recovery bouts Time: Duration to equal total metabolic equivalents of HCT for 25 min at 80% Wpeak Modality: Cycle ergometer	Responses to acute bout: All but one subject were able to achieve the target exercise duration. Overall, they were able to maintain their target HR range. The mean HR attained as percentage of target was 99.9% (HCT), 99.8% (MCT), and 89.6% (HIIT). Perceived leg fatigue was significantly less in MCT than in the other groups. Mean HR attained were similar between all groups. Response to 12-week intervention: See feasibility and tolerability.	Compliance: Mean attendance not significantly different between groups, means were 70.1–81.9% Mean 12-week adherence to target intensity was significantly lower in HIIT (49%) compared to other groups HCT (85.6%) and MCT (85.4%). In acute session, mean HR attained as a percentage of target was 99.9% for HCT, 99.8% for MCT, and 89.6% for HIIT.
Rodriguez et al. (2016) [84]	Frequency: 3×/week for 8 weeks Interval: 8 intervals at 70–80% Wpeak for 2 min Rest: recovery at 40–50% Wpeak for 3 min Modality: Cycle ergometer	Cardiac autonomic function (measured by heart rate recovery) was improved in both MCT and HIIT. After the interventions, there was a significant increase in VO_2peak_ by 17%, and Wpeak by 18% in both groups. Both the chronotropic response and heart rate recovery improved by 45% and 26% respectively. The change in resting HR was only significantly different in the MCT group.	Not given
Uc et al. (2014) [85]	Frequency: 3×/week for 6 months Intervals: 3-min intervals at 80–90% HR_max_ Rest: 60–70% HR_max_ for 3 min Time: 15 to 45 min (progressive) Modality: Walking	Aerobic fitness and motor function were significantly and similarly improved in both groups among those who completed the intervention, per VO_2max_ (mL/min/kg ± SD = 1.65 ± 2.90) and 7-min walk time (s ± SD = − 0.66 ± 1.06). Measures of executive function, fatigue, depression, and quality of life were also improved across all completers with no difference between interventions. MCT in individual settings demonstrated similar improvements with better retention, adherence, and safety compared to HIIT.	Dropouts: 3 participants in the HIIT group dropped out due to exercise-related knee pain (reversible with rest and conservative measures). None in the continuous group. Compliance: 81% completed study with 83.3% avg. attendance, exercising at 46.8% HRR. % of required sessions completed: 81.4% of MCT, 73.0% of HIIT AEs: No serious adverse events were reported

*HIIT* high-intensity interval training; *HCT* high-intensity continuous training; *MCT* moderate-intensity continuous training; *RPE* rating of perceived exertion; *HR* heart rate; *PPO* peak power output; *W* work; *VO*_*2*_ volume of oxygen consumption; *HRR* heart rate reserve

### Non-clinical Populations

Studies including non-clinical populations made up the largest subgroup in this scoping review (*n* = 30) and included people who were sedentary or active at baseline and were typically free of significant disease or had well-controlled medical conditions (Table 2) [18–46, 87, 88]. Studies in this category were more likely to use SIT as the high-intensity exercise intervention (*n* = 7) and the most common exercise modality was cycling (*n* = 22). In HIIT studies, the interval durations were most commonly 1 or 4 min. Notably, a large number of these studies examined the effects of HIIT after only a single session (*n* = 11). Where HIIT was compared to MCT after a single training session in this group, HIIT generally caused more significant attenuation of flow-mediated dilation (FMD) than MCT [20, 46]. Nederveen et al. [37] found that HIIT induced greater satellite cell response to exercise than MCT while both had similar completion rates. Stockwell et al. [40] reported that across a single session, HIIT was generally preferred over MCT by participants.

Six studies examined HIIT vs. MCT over longer training periods [21, 23, 24, 28–30]. Here, HIIT was found to be both tolerable and feasible and had a greater impact on VO_2peak_ [21, 30], ejection fraction, and insulin resistance compared to MCT [28]. Kim and colleagues found that arterial stiffness improved only in MCT and not after HIIT [29]. In regards to memory and cognition, high-interference memory was found to be improved after HIIT but not after MCT [30]. Brown et al. (2021) found there to be no direct impact of exercise (HIIT or MCT) on cognition [21].

### Cardiovascular Disease

12 studies examined the impacts of HIIT in people with known cardiovascular disease including coronary artery disease, hypertension, stroke, abdominal aortic aneurysms, and peripheral arterial disease **(**Table 3) [47–58]. The most common training intervention in these populations was a single HIIT session (*n* = 7). Four of the study protocols used short intervals lasting 1 min in duration, alternating with rest periods also lasting 1 min. Six studies in this group measured interval intensity by power or work output. The most common exercise modality used was cycling (*n* = 8).

Where studies examined outcomes of acute HIIT sessions compared to acute MCT sessions, some conflicting results were found. Bailey et al. [47] found that HIIT causes a decrease in flow-mediated dilation (FMD) while MCT causes this to increase. Currie et al. found that both HIIT and MCT cause an increase in FMD [48] and similar findings were reported by the same authors in longer training interventions [49]. HIIT was found to induce greater hypotensive effects compared to MCT by both dos Santos et al. [50] and Sosner et al. [56]. One study reported that participants in this category demonstrated a preference for HIIT over MCT [50]. Across population cohorts, there were mixed results as to the effects of HIIT compared to continuous training on blood pressure. Blood pressure was shown to decrease from baseline to 1 h after interval training, up to 10 mmHg in systolic blood pressure, though it was variable whether or not this was statistically greater than a MCT group [50, 56, 59]. Reichert et al. had participants with hypertension complete 28 weeks of HIIT or HCT [55]. Here, it was found that systolic and diastolic blood pressures were decreased in both training groups but that the diastolic decrease was greater in the continuous training group [55].

### Cardiac Disease

Of the 9 studies which employed HIIT in patients with heart failure [59–67], the majority (*n* = 6) used the “4 × 4” HIIT protocol (Table 4). This intervention includes 4 bouts of long, 4-min intervals at high intensity, interspersed with 3-min rest periods. Every study in this population involved a non-acute training session of 4 to 12 weeks in duration, with 12 weeks being the most common (*n* = 7). Cycle ergometers and treadmills were the most common means of exercising in this group.

In studies where HIIT was compared to MCT, HIIT was found to result in a larger reduction in blood pressure [59, 62] and had a greater or similar improvement in VO_2peak_ [59–62, 67]. HIIT was also found to have similar effects compared to MCT on left ventricular end-diastolic function and left ventricular end-diastolic diameter [60, 62] and on metabolic improvements [62].

### Metabolic Disease

Studies considered to be metabolic diseases included populations with type 2 diabetes mellitus (T2DM) [71–73, 75], pre-diabetes [68, 69], and obesity [70, 74] (Table 5). Similar to protocols used in the cardiac disease grouping, these interventions tended to have variable durations of training, ranging from 2 to 16 weeks. The most common duration of training was for 8 weeks (*n* = 3). A treadmill-based exercise was the most common modality in these populations (*n* = 4).

In studies comparing HIIT to MCT, the results can be divided by length of intervention. In studies of 8 weeks duration, little difference was seen between HIIT and MCT in terms of change in body composition, metabolic profile, cardiovascular risk, and aerobic capacity [70, 71]. In studies lasting 12 and 16 weeks, HIIT was seen to have a larger decrease compared to MCT in total abdominal and visceral fat mass [73] and in BMI and metabolic profile [75]. HIIT was found to be similarly enjoyed and tolerated compared to MCT [70, 71]. Maillard et al. [73] observed that over a 16-week intervention, both HIIT and MCT groups had a similar decrease in whole-body fat mass of (mean ± SD) − 2.5 ± 1.3% in the HIIT group, and − 3.2 ± 1.2% in the MCT group. Notably, after this longer intervention, there was only one reported dropout from the MCT group for personal reasons.

### Other Clinical Populations

Other studies examined HIIT in populations with chronic obstructive pulmonary disease (COPD) [83, 84], Parkinson’s disease [78, 85], Alzheimer’s disease [79], cancer [76, 77, 82], knee osteoarthritis [80], and systemic sclerosis [81] (Table 6). Cycling was the most common exercise modality in these groups (*n* = 8). Interventions with COPD populations lasted between 8 and 12 weeks. In this population, HIIT was found to have a similar impact on cardiac autonomic function, aerobic fitness, tolerability, and compliance compared to MCT [83, 84]. In neurocognitive disease states, HIIT was found to have a variable impact on cognitive scores and functional abilities compared to MCT, with two studies showing no effect [79, 85] and one showing positive effects [78]. Notably, Uc et al. [85] studied a 6-month HIIT intervention in participants with Parkinson’s disease. This long-duration study demonstrated that both HIIT and MCT improved VO_2max_ in participants by 1.65 [2.90] mL/min/kg (mean [SD]), though this may not be clinically meaningful. This study did demonstrate that compliance across both groups was fairly high: 81% of participants completed the study and the percentage of sessions completed in each group was 81.4% (MCT) and 73.0% (HIIT). Over the 6-month training period, no serious adverse events were reported. Another longer intervention of 16 weeks with participants with mild Alzheimer’s disease by Hoffmann et al. [79] showed that 76% of the HIIT group attended more than 80% of the sessions and 78% of the participants exercised at over 70% of their HR_max_. There were 35 adverse events and 7 serious adverse events reported during this study. The adverse events suspected to be related to the intervention included musculoskeletal problems, dizziness, and one episode of atrial fibrillation. In this study, there was no change from baseline in cognitive scores, quality of life, or activities of daily living in the HIIT group. Across all studies in this diverse range of clinical populations, HIIT was generally found to have a high exercise adherence [76, 79–82, 85]. Where it was compared to MCT, attendance ranged from 70.1–94% of sessions and was similar between treatment groups [83, 85].

## Discussion

This scoping review characterized existing literature on HIIT in older adults including exercise protocols administered, main outcomes, and feasibility and safety. The purpose of this review was to assist in knowledge translation for clinicians, as well as to recommend areas of further research. In brief, 69 studies involving 3243 individuals belonging to both clinical and non-clinical populations were included in this review. The main findings and recommendations of this scoping review are discussed below.

### HIIT Protocols Used for Older Adults

The studies in this review used a range of HIIT protocols which varied in frequency and duration (single sessions to 6-month interventions), length of interval (seconds to minutes), intensity of interval (70% HR_peak_ to supramaximal intensity), and modality. In spite of the lack of consensus on which protocols are optimal for maximal benefits, some protocols were seen more commonly with certain groups. The “4 × 4” protocol (*n* = 15), which is 4 bouts of 4-min intervals interspersed with 3-min rest—usually 3×/week for at least 4 weeks—was most popular for use in subjects with heart failure [59, 60, 62–64, 66], but was also used in non-clinical populations [28–30, 33, 45, 46, 87], peripheral arterial disease [52], T2DM [71], and in colorectal cancer survivors [77]. It has been reported previously that longer work intervals and higher weekly energy expenditure may be more effective in increasing adaptations such as VO_2max_ and cardiac function in populations with heart failure [89]. Protocols of alternating 1-min high intensity and 1-min rest for 10–12 intervals (“10 × 1” protocol) were the next most common in this age group (*n* = 9). In all but one study, these 10 × 1 protocols were used in single training sessions. As such, it is difficult to measure the effects as well as tolerability and feasibility of longer interventions in this age group. SIT was also commonly seen (*n* = 10). With the exception of one study in participants with limited cutaneous systemic sclerosis [81], all studies using SIT were in healthy populations.

Cycle ergometers were the most commonly used means of facilitating HIIT in this age group, followed by treadmills. These methods are likely to be among the most accessible to individuals who want to incorporate HIIT into their exercise routines, either at a gym or by biking or walking/running outdoors. Less commonly used methods of achieving aerobic exercise included aquatic training, non-weight-bearing all-extremity ergometers, and recumbent steppers. Although these modalities may seem less accessible to the average older adult, these alternative modalities are important to continue to include in future research as they may allow for greater participation among older adults with mobility limitations.

### HIIT and Aerobic Fitness

The most common outcome measured in the included studies was a change in VO_2peak_ as a measure of aerobic fitness. Across clinical and non-clinical populations, HIIT was shown to increase VO_2peak_ more than the control [21, 27, 28, 30, 34, 45, 52, 60, 62–64, 67, 71, 81, 85]. Conversely, one study found no change between HIIT and control [57]. Where change in VO_2peak_ or PPO was compared between age cohorts, HIIT was shown to increase aerobic fitness similarly across both younger and older age groups [41, 43, 87]. When directly compared between two groups in one study, HIIT was also shown to increase aerobic capacity between a healthy cohort and a cohort with heart failure [65]. The evidence becomes conflicting when comparing change in VO_2peak_ between HIIT and traditional endurance training. Most authors found that both HIIT and MCT had similar effects on VO_2peak_ [30, 49, 60, 62, 67, 70, 71, 81, 84, 85]. However, some studies found HIIT to be superior [21, 28, 59, 61, 82]. These studies were diverse in clinical populations as well as in HIIT protocols, and as such, it remains unclear which factors are most likely to correlate with maximum success of the intervention.

These results are suggestive that HIIT can be an effective means of improving aerobic fitness in older adults, and that it may confer a small advantage over traditional endurance training. This is consistent with the existing literature on non-elderly adults which showed that HIIT may have a small benefit compared to MCT on improving VO_2peak_, but that this improvement is likely to be increased by longer intervals and greater work-to-rest ratios, and in older or less-fit subjects [90, 91].

### HIIT and Vascular Outcomes

The most common outcomes of vascular function included in the literature were blood pressure measurements (systolic, diastolic, 24-h ambulatory) and FMD, and these yielded mixed results. A decrease in blood pressure was larger in HIIT when compared to RT [22, 38] and when compared to MCT [38]. In one study, there was no change seen in ambulatory blood pressure in either MCT or HIIT group [62]. In studies measuring FMD, results were variable. In non-acute studies, no change was seen in FMD between MCT and HIIT [49, 59, 67]. In acute studies, changes in FMD varied depending on the cohort’s fitness [20], sex [46], and were conflicting whether HIIT attenuated, increased, or had no impact on FMD compared to MCT [47, 48]. One study examined change in arterial stiffness, and this was only seen to improve after MCT [29].

### HIIT and Cardiac Function

Measures of cardiac function were examined in populations with heart failure and in one study of non-clinical populations. HIIT was found to increase left ventricular end-diastolic diameter compared to control either similar to MCT [60] or to be superior to MCT [28, 59]. Conversely, HIIT was not seen to impact cardiac function in three studies [62, 66, 67].

### HIIT and Metabolic Factors

Metabolic factors were most often measured in populations with obesity, T2DM, and pre-diabetes, as well as in non-clinical populations. In these studies, glycemic control was seen to improve compared to controls and was superior compared to MCT [28, 72] or to be similar to MCT [62]. Most studies using body fat or body composition as an outcome measure, including a study in participants with knee osteoarthritis, did not find that interval or continuous training resulted in significant changes [70, 72, 80]. Maillard et al. however, observed that HIIT resulted in a larger decrease in total abdominal and visceral fat mass, but not whole-body fat compared to MCT [73]. Conversely, Hwang et al. noted that MCT had a larger decrease in body fat than HIIT [71]. Regarding lipid markers and cholesterol changes, Boukabous et al. found that HIIT and MCT had resulted in similar improvements [70] whereas no change was seen by Hwang et al. [71].

In non-elderly adults, two systematic reviews and meta-analyses by Keating et al. [92] and Wewege et al. [93] compared the effect of HIIT or SIT to MCT on changes in body adiposity. Both studies found interval and continuous training to result in similarly decreased total body fat over a range of intervention durations. This is similar to the findings of this scoping review which identified that the HIIT interventions ≤ 8 weeks in duration had a similar effect on body composition and adiposity compared to MCT. The findings of this scoping review, however, suggest that longer interventions in older adults may reveal higher efficacy of HIIT compared to MCT.

### HIIT and Neurocognitive Decline

Neurocognitive decline is prevalent among adults of advanced age and is an important contributor to reduced health and quality of life [94]. Three studies included in this review used HIIT in populations with known cognitive decline: two in Parkinson’s disease [78, 85], and one in mild Alzheimer’s disease [79]. Two more examined cognitive outcomes in non-clinical populations [21, 33]. Of these studies which measured cognitive outcomes in both HIIT and MCT, in some, HIIT was not found to be associated with any improvement on cognitive scores [21, 33, 79]. Conversely, in one study, it was also found to improve immediate auditory memory similar to MCT and improve both attention and sustained attention greater than MCT [78]. In Northey et al.’s study on breast cancer survivors, cognitive performance was also measured, and HIIT was seen to have a moderate to large positive but statistically insignificant effect compared to MCT and control [82].

### HIIT and Osteoarthritis

Only one study examined HIIT in osteoarthritis, and it showed that HIIT had higher adherence than the MCT group (94% compared to 88%) and that both groups had improvements in health-related quality of life scores. Though neither intervention resulted in a change in body composition, HIIT was shown to improve physical function similar to or greater than MCT [80]. Though this is only one small study, these results are supportive of HIIT use in this population and further research should be pursued.

### Feasibility and Tolerability of HIIT

One of the aims of this review was to examine whether HIIT protocols are feasible and/or tolerable in the older adult population. The HIIT protocols employed in the included studies were generally well tolerated, had few adverse events which were comparable to those of MCT interventions, and had good attendance which was also comparable to attendance for MCT interventions. One exception to this was the 6-month study by Uc et al. [85], where three participants in the HIIT group dropped out due to exercise-related knee pain. After initially randomizing participants to HIIT or MCT, later cohorts in this study were allocated to MCT only. In several studies, HIIT was deemed to be as or more enjoyable by participants than MCT [40, 50, 81].

### Recommendations and Future Directions

Of the studies included in this review, only 57% examined the impact of HIIT in clinical populations and many prevalent chronic diseases among the elderly were not represented at all. This percentage is low when considering that in some countries, more than 85% of people over the age of 65 are reported to live with at least one chronic medical condition [95, 96] and a significant proportion of those may have multiple medical comorbidities [95, 97]. Cardiovascular diseases, including chronic ischemic heart disease, CHF, and arrhythmia are the leading causes of death in older adults followed by cancer [94]. These were present in the literature, although given their significant mortality, they should be further studied. Additionally, the most common chronic diseases in adults over 85 years of age are hypertension, osteoarthritis, T2DM, and osteoporosis [94]. Notably, osteoarthritis was represented in only one small study and clinical groups with osteoporosis were absent from the available literature altogether. Other prevalent conditions among the elderly include frailty and depression [94]. More studies on HIIT in these populations will be important additions to the existing knowledge of its impact and tolerability.

There is still no consensus on which HIIT protocol is most effective in older adults. Though some HIIT protocols appeared in the literature more often, namely the “4 × 4” and the “10 × 1” protocols, there was still much variation in frequency and duration of training, clinical population studied, and outcomes measured. As such, it was very difficult to directly compare which of these common training methods are most likely to induce training results. Further research should compare these and other HIIT and SIT protocols for clinical outcomes as well as feasibility and tolerability.

The current Physical Activity Guidelines for Americans were established for adults over 65 years of age with emphasis on preserving or improving functional independence and quality of life [5]. These goals are not adequately represented by the measured outcomes of the included studies and as such, further research is needed.

## Conclusion

In summary, as the global population continues to age, early research on the impact of aerobic HIIT in older adults suggests that this training method is generally well-tolerated, feasible, and may confer many health advantages to this population. The majority of studies included in this review were in non-clinical populations and compared HIIT to MCT or to a control group. These studies are still few in number, small in sample sizes, and do not yet represent the scope of chronic diseases which are nearly ubiquitous in this age group. As such, more research is needed on the effects, feasibility, and tolerability of HIIT in these clinical populations in order to support our aging population with best health practices and exercise recommendations.

## Additional File

**Additional file 1.**

## Data Availability

Data will be made available upon reasonable request.

## Abbreviations

HIIT: High-intensity interval training
RT: Resistance training
MIT: Moderate-intensity interval training
MCT: Moderate-intensity continuous training
VT_2_: Anaerobic threshold
VT_1_: Aerobic threshold
%PPO: Percentage of peak power output
SIT: Sprint interval training
HRR: Heart rate reserve
HR: Heart rate
VO_2_: Volume of oxygen consumption
T2DM: Type 2 diabetes mellitus
COPD: Chronic obstructive pulmonary disease
FMD: Flow-mediated dilation
