# Supplementary material for: High-Intensity Interval Training in Older Adults: a Scoping Review
Source: Sports Med Open. 2021 Jul 19;7:49. doi: 10.1186/s40798-021-00344-4 (PMC8289951; doi:10.1186/s40798-021-00344-4)
Supplement: Supplementary file 1 — Additional file 1. [file 40798_2021_344_MOESM1_ESM.pdf]

AUTHOR DECLARATION FORM

At submission, **EVERY AUTHOR** listed in the manuscript must **READ** and **COMPLETE** the following statements on: (A) Authorship Responsibility, (B) Authorship Criteria, (C) Authorship Contribution, (D) Funding Disclosures, (E) Contributor Disclosures/Acknowledgments, and (F) Conflicts of Interest Disclosures.

It is important that you return this form as early as possible in the publication process. **EVERY AUTHOR MUST COMPLETE AN INDIVIDUAL COPY OF THE FORM, AND EVERY SECTION OF THE FORM MUST BE COMPLETED.** We will **NOT** consider your manuscript for publication until every author has completed the form and returned it to us.

Your name (please print): Katie Marriott E-mail: cmarriott2021@meds.uwo.ca

Journal name: Sports Medicine Corresponding author: Dr. Robert Petrella

Manuscript title: HIT in Older Adults: A Scoping Review

A. AUTHORSHIP RESPONSIBILITY

☒ I certify that **ALL** of the following statements are correct **(PLEASE CHECK THE BOX)**.

- The manuscript represents valid work; neither this manuscript nor one with substantially similar content under my authorship has been published or is being considered for publication elsewhere (except as described in the manuscript submission); and copies of any closely related manuscripts are enclosed in the manuscript submission; ***AND***
- For manuscripts with more than one author, I agree to allow the corresponding author to serve as the primary correspondent with the editorial office and to review and sign off on the final proofs prior to publication; or, if I am the only author, I will be the corresponding author and agree to serve in the roles described above.
- For manuscripts that are a report of a study, I confirm that this work is an accurate representation of the trial results.

B. AUTHORSHIP CRITERIA

To fulfil all of the criteria for authorship, every author of the manuscript must have made substantial contributions to **ALL** of the following aspects of the work:

- Conception and planning of the work that led to the manuscript or acquisition, analysis and interpretation of the data, or both; ***AND***
- Drafting and/or critical revision of the manuscript for important intellectual content; ***AND***
- Approval of the final submitted version of the manuscript.

☒ I certify that I fulfill **ALL** of the above criteria for authorship **(PLEASE CHECK THE BOX)**.

C. AUTHORSHIP CONTRIBUTION

I certify that I have participated sufficiently in the work to take public responsibility for **(PLEASE CHECK 1 OF THE 2 BOXES BELOW)**:

☐ Part of the content of the manuscript; ***OR***
☒ The entire content of the manuscript.

D. FUNDING DISCLOSURES

PLEASE CHECK 1 OF THE 2 BOXES BELOW:

☐ I certify that no funding has been received for the conduct of this study and/or preparation of this manuscript; ***OR***
☒ I certify that all financial and material support for the conduct of this study and/or preparation of this manuscript is clearly described in the Compliance with Ethical Standards section of the manuscript.

Some funding organizations require that authors of manuscripts reporting research deposit those manuscripts with an approved public repository.

☐ Please check here if you have received such funding.

E. CONTRIBUTOR DISCLOSURES

All persons who have made substantial contributions to the work reported in the manuscript (e.g. data collection, data analysis, or writing or editing assistance) but who do not fulfill the authorship criteria **MUST** be named with their specific contributions in the Acknowledgments section of the manuscript. Groups of persons who have contributed may be listed under a heading such as 'Clinical investigators' and their function described. Because readers may infer their endorsement of the manuscript, all persons named in the Acknowledgments section **MUST** give the authors their written permission to be named in the manuscript.

☒ I certify that all persons who have made substantial contributions to this manuscript but who do not fulfill the authorship criteria are listed with their specific contributions in the Acknowledgments section in the manuscript, and that all persons named in the Acknowledgments section have given me written permission to be named in the manuscript.

F. CONFLICT OF INTEREST DISCLOSURES

A conflict of interest exists when professional judgment concerning a primary interest (such as patients' welfare or the validity of research) may be influenced by a secondary interest (such as financial gain or personal rivalry). A conflict of interest may arise for authors when they have a financial interest that may influence – probably without their knowing – their interpretation of their results or those of others. We believe that to make the best decision on how to deal with a manuscript we should know about any such conflict of interest that the authors may have. We are not aiming to eradicate conflicts of interests – they are almost inevitable. We will not reject manuscripts simply because the authors have a conflict of interest, but we will publish a declaration in the manuscript as to whether or not the authors have conflicts of interests.

All authors **MUST** complete the following checklist:

**Category of potential conflict of interest**	If you have had any of the listed relationships with an entity that has a financial interest in the subject matter discussed in this manuscript, please check the appropriate "Yes" box below and provide details. If you do not have a listed relationship, please check the appropriate "No" box. When completing this section, please take into account the last 36 months through to the foreseeable future.		
	**No (√)**	**Yes (√)**	**Details**
Employment	x		
Grant received/grants pending	x		
Consulting fees or honorarium	x		
Support for travel to meetings for the study, manuscript preparation or other purposes	x		
Fees for participation in review activities such as data monitoring boards, etc	x		
Payment for writing or reviewing the manuscript	x		
Provision of writing assistance, medicines, equipment or administrative support	x		
Payment for lectures including service on speakers bureaus	x		
Stock/stock options	x		
Expert testimony	x		
Patents (planned, pending or issued)	x		
Royalties	x		
Other (err on the side of full disclosure)	x		

Every author **MUST** complete option 1 or option 2 as appropriate below. If you answered "Yes" to any of the questions relating to financial conflicts of interests in the table above (or if you wish to disclose a non-financial conflict of interest), you **MUST** write a suitable statement in the box below and include this statement in the Compliance with Ethical Standards section of the manuscript.

☒ I have no conflicts of interest to declare; ***OR***

☐ The following statement regarding conflicts of interest and financial support for conduct of this study and/or preparation of this manuscript is to be published in the Compliance with Ethical Standards section of the manuscript:

Declaration: I certify that I have fully read and fully understood this form, and that the information that I have presented here is accurate and complete to the best of my knowledge.

Your name (please print): Katie Marriott

Signature (please **HAND-WRITE**): Katie Marriott

Date: September 12, 2020
